# Alternative mammalian strategies leading towards gastrulation: losing polar trophoblast (Rauber's layer) or gaining an epiblast cavity

**DOI:** 10.1098/rstb.2021.0254

**Published:** 2022-12-05

**Authors:** Peter L. Pfeffer

**Affiliations:** School of Biological Sciences, Victoria University of Wellington, Kelburn Parade, Wellington 6010, New Zealand

**Keywords:** polar trophoblast, epiblast, amniotic cavity, eutherians, Rauber's layer

## Abstract

Using embryological data from 14 mammalian orders, the hypothesis is presented that in placental mammals, epiblast cavitation and polar trophoblast loss are alternative developmental solutions to shield the central epiblast from extraembryonic signalling. It is argued that such reciprocal signalling between the edge of the epiblast and the adjoining polar trophoblast or edge of the mural trophoblast or with the amniotic ectoderm is necessary for the induction of gastrulation.

This article is part of the theme issue ‘Extraembryonic tissues: exploring concepts, definitions and functions across the animal kingdom’.

## 1. Introduction

**Figure 1.**
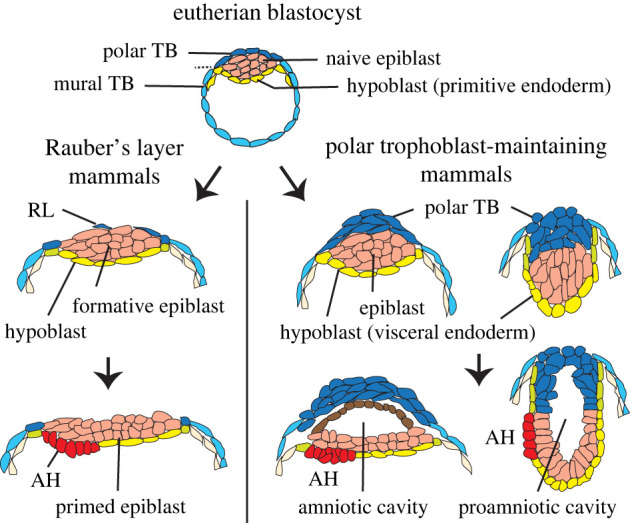
Developmental trajectories in eutherian mammals beyond the blastocyst. In Rauber's layer (RL) mammals the polar trophoblast overlying the epiblast disintegrates (left) while in others it proliferates (right). The epiblast matures from a pluripotent naive state via an epitheloid/formative to a primed epithelial or stratified-epithelial state. In polar trophoblast (TB)-maintaining mammals the epiblast develops a cavity termed the amniotic cavity or, if the roof (shown in brown) is lost/absent, a proamniotic cavity. Part of the hypoblast thickens to form the anterior hypoblast (AH), also termed anterior visceral endoderm, which secretes inhibitors that pattern the epiblast.

August Rauber noticed in 1875 that the epiblast of early rabbit embryos was covered by a thin layer of cells that subsequently dissolves [1]. He termed this the ‘Deckschicht’, but it was Koellicker who realised that these cells were trophoblast and who named the layer after its discoverer [2]. That notation has henceforth been used in species where the trophoblast overlying the epiblast is lost (figure 1). However, mentioning ‘Rauber's layer’ may well meet with incomprehension even among developmental biologists. This can be explained by the brief existence and the perceived inconsequential nature of this tissue, even though half of all mammalian species possess it. I will here place Rauber's layer in a new context, paradoxically emphasizing the importance of its disappearance rather than its existence.

## 2. Events leading to the formation of polar trophoblast

**Figure 2.**
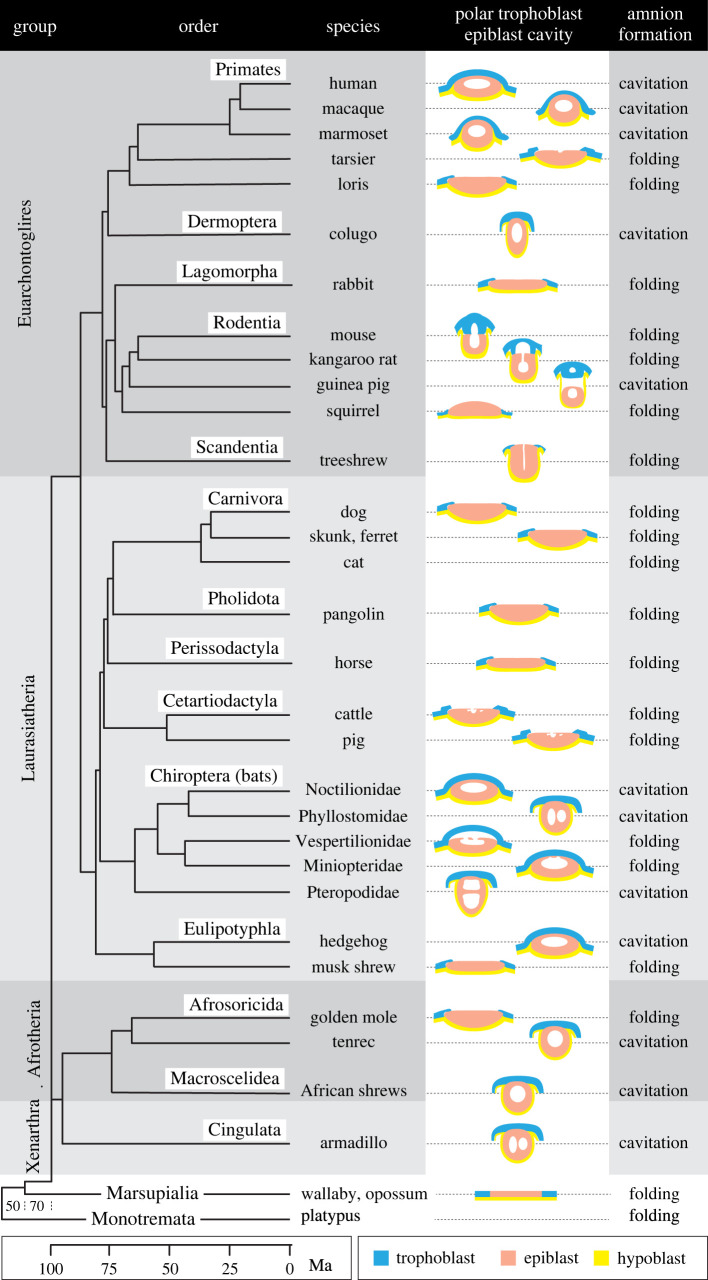
Polar trophoblast fate, epiblast cavity formation and mode of amnion formation in mammals. Distribution of Rauber's layer and polar trophoblast-retaining species across 14 orders and among major lines within the orders. Epiblast cavitation is indicated – where the cavity extends into the trophoblast it is termed a proamniotic cavity as it will not contribute to the amnion. Amnion formation is indicated as ‘folding’ where the amniotic ectoderm forms by folding/ingrowth from the edge of the epiblast disc at gastrulation. If the epiblast cavity expands to eventually form the amnion, this is indicated as ‘cavitation’. Cladogram adapted from [6] and [7]. References for embryological data: human [8], macaque monkeys [9], marmoset (New World monkey) and loris [10], tarsier [11], colugo [12], rabbit [13], mouse [14], kangaroo rat [15,16], guinea pig [17], squirrel [15,18], treeshrew [19], dog [20,21], skunk [22], ferret [23], cat [20], pangolin [24], horse [25], cattle [26], pig [27], Noctilionidae: lesser bulldog bat [28], Phyllostimidae: short-tailed fruit bat [29], Vespertiliionidae: little brown bat [30], common noctule [31], Miniopteridae: natal clinging bat [32], Pteropodidae: cynopterine fruit bat [33], hedgehog [34], musk shrew [35], golden mole [36], tenrec [37], African shrews: elephant shrew [38], North African jumping shrew [39], armadillo [40], marsupials and monotremes [4]. Ma, millions of years ago.

After fertilization, the proteinaceous zona pellucida surrounding the egg hardens and the mammalian zygote undergoes successive rounds of cleavage, that is, cell divisions without concomitant growth. In monotreme (platypus and echidna) and marsupial mammals, the developing embryo is further enclosed by a permeable egg shell layer and the dividing cells coalesce and then grow along the inside surface of these containing layers to eventually form a hollow single-layered spherical epithelium. When this sphere consists of several hundred cells, a patch of cells destined to form the pluriblast can be identified [3]. It is the pluriblast that will give rise to the fetus as well as most extraembryonic membranes [4]. During further development the pluriblast develops into (i) the epiblast, which is the progenitor layer of the embryo proper, and (ii) the underlying hypoblast (also termed primitive endoderm), which mainly gives rise to the yolk sac. The non-pluriblast (major) part of the hollow spherical embryo is the trophoblast, which will form the outermost layer of the chorion [5]. Notably, in these two ancestral branches of mammals, the trophoblast does not cover the pluriblast, but abuts it in a planar fashion, creating a trophoblast–pluriblast epithelial border that is subsequently resolved into a trophoblast–epiblast border (figure 2).

The third branch of mammals, the placental mammals, or eutherians, diverged over the last 100 Myr into four superordinal groups (figure 2), the oldest branches being the Xenarthrans (two extant orders; e.g. armadillo) and Afrotherians (six orders; e.g. elephants, elephant shrew, tenrec). The most recent branching led to the populous Laurasiatherians (six orders including carnivores, most farm animals and bats) and Euarchontoglirians (five orders including primates, rodents, rabbits and cologu). Common and unique to all 19 orders is the formation of the blastocyst-embryo, a spherical structure consisting of a unilaminar trophoblast epithelium surrounding a fluid-filled cavity that is partially filled with a clump of pluriblast-like cells termed the inner cell mass (ICM). Importantly, as the ICM cluster is located eccentrically within the blastocyst cavity, two types of trophoblast can be distinguished: the polar trophoblast overlying the ICM and the mural trophoblast that does not (figure 1). This eutherian-specific combination of tissues is fundamentally different from that in monotreme and marsupial mammals in that a novel cell population and interactive interface have been created, namely the *polar trophoblast* and the interaction of this layer with the entire outermost (dorsal) ICM surface. As we shall see, this novel configuration has led to alternate developmental strategies.

Before following the polar trophoblast fate further, it is worth noting that this trophoblast arose because of the internalization of the pluriblast. Epiblast internalization is likely to have been an adaptation to cope with the loss of the amniote eggshell [41], which in non-placental mammals is still present at early embryonic stages. Loss of the shell would result in more extensive and intimate cell contact between conceptus and maternal endometrium, which benefits efficient nutrient and oxygen transfer, but also requires differentiation of the interacting outside cells. Internalization of the pluriblast cells thus has two advantages. First, the inside cells would have been freed from the differentiative and epithelial polarization requirements placed on outer cells, allowing inside cells to follow alternative (viz. pluripotent/ICM) fates. Second, having only one cell type on the outside would minimize the number of antigens presented to the maternal immune system.

## 3. Alternative trajectories of the polar trophoblast

Somewhat unexpectedly, once formed, the fate of the polar trophoblast diverges dramatically among eutherians (figures 1 and 2). In roughly half the orders, the polar trophoblast layer is lost and hence is termed Rauber's layer. In the other species, the polar trophoblast keeps proliferating and is sometimes variably referred to as extraembryonic ectoderm (murines) or cytotrophoblast (primates, bats). Both types of polar trophoblast fates are seen in species of all four superordinal groups (figure 2). Furthermore, in four mammalian orders (rodents, primates, Eulipotyphlians (hedgehog versus true shrew), Afrosorcidians (tenrec versus golden mole)), a mixture of Rauber's layer and polar trophoblast-retaining species exist (figure 2). Together this suggests that a switch from one state to the other has occurred repeatedly during evolution. It is likely that the Rauber's layer (loss) condition is the less derived one based on the observation that within the primates and rodents, species in the more primitive/ancestral branches lose the polar trophoblast, as do their closest sister-orders, colugos and rabbits, respectively [20].

Preceding the bifurcation in polar trophoblast fate, the underlying ICM cells differentiate into either NANOG- and SOX2-expressing epiblast cells or GATA6-positive hypoblast cells as shown for mice [42], rabbits [43], cattle [44], pigs [45], marmoset monkeys [46] and humans [47]. In these species, the hypoblast and epiblast progenitors are initially interspersed within the ICM, but soon segregate into an ellipsoid of epiblast cells touching the polar trophoblast on the outside-facing surface, and a layer of hypoblast cells covering the blastocyst cavity-facing inner side (figure 1) [48]. In elephant shrews, tenrecs and the lesser bulldog bat, ICM formation is delayed and hypoblast may be formed without a sorting phase, but no expression analyses have been undertaken [28,37,49]. Either way, in all cases the polar trophoblast initially overlays the early or ‘naive’ epiblast.

### (a) Rauber's layer species

At the naive epiblast stage, electron micrographs reveal a close apposition of polar trophoblast (Rauber's layer) cells with the underlying epiblast in Rauber's layer species such as the horse [25], pig [50], cattle [51] and rabbit [13]. While a basement membrane (Reichert's membrane) is formed in regions where the mural trophoblast contacts the expanding hypoblast layer, no such matrix is formed between Rauber's layer and the epiblast. Thus Rauber's layer is exposed to signals from the epiblast, but what the signals are that contribute to its disappearance, is not known. Potentially fibroblast growth factor (FGF) signalling could be involved, because in pigs, Rauber's layer expresses more FGF receptor 2 (FGFR2) than the rest of the trophoblast [52].

The mode and mechanism of Rauber's layer disappearance appears not to be conserved. Prior to its disappearance, the layer thins in some species, possibly because of stretching forces arising as the underlying epiblast morphs from a spheroid to a flatter shape while actively proliferating. While this may contribute to the multiple breaks appearing in Rauber's layer as described for the rabbit [13] and horse [25], such a mechanism would not be universal, as for example in cats, Rauber's layer is lost before any change is detected in epiblast surface area [20]. Similarly, in cattle epiblast, expansion occurs in all three dimensions, while the proliferation rate is equal in Rauber's layer and the epiblast, thus precluding the possibility of the epiblast outgrowing the trophoblast [53]. In the horse [25], cattle [53] and rabbit [13] apoptosis is involved, while in pigs there is evidence for a combination of apoptosis and autophagocytosis [27]. Subsequently, polar trophoblast cell remnants are phagocytosed by the underlying epiblast cells in the rabbit [13], horse [25] and pig [27]. By contrast, in the skunk (order Carnivoria), peripheral displacement rather than cell death was suggested to underly Rauber's layer disappearance [22].

The mode of Rauber's layer loss varies too. In rabbits and horses, multiple breaks appear all over Rauber's layer with surviving cells scattered across the expanding epiblast for a while [13,25]. In pigs and cattle, asymmetric holes develop that then merge, transiently leaving coalescent patches of polar trophoblast still adhered to epiblast [26,27,53]. In pigs, some of these Rauber's layer cells are lost together with (and while still adhering to) the outside-most (dorsal) cells of the epiblast [27]. It is likely that these different modes of Rauber's layer loss reflect the relative adhesion of polar trophoblast to themselves in comparison to epiblast cells.

What is conserved however, is the maintenance of the embryo's overall epithelial integrity. In other words, at the time that Rauber's layer is lost, naive epiblast cells, particularly those closest to the inside, start forming junctional contacts with neighbouring epiblast cells as well as with the mural trophoblast cells located at the edge of the epiblast mass. This has been shown in rabbits [13], cattle [51,54], horses [25] and pigs [27,55]. These close contacts are the first indication of epiblast epithelialization and preserve the turgidity of the blastocyst as the junctional integrity of Rauber's layer is lost. Hence an inward collapse of the blastocyst is prevented, and a barrier between the blastocyst cavity and the outside (maternal) environment is maintained [50]. The incumbent epithelialization process represents the transition of naive to a ‘formative’ [56] or ‘epitheloid’ epiblast [57]. Further cell polarization leads to the *embryonic ectoderm* epithelium or ‘primed epiblast’ (primed for embryonic lineage determination; [58]), which is likely to be the ancestral amniote pluriblast-derived configuration for gastrulation to proceed [57]. Morphologically, these epithelialization changes are accompanied by the conversion of the ellipsoid early naive epiblast to a planar or indented disc-shaped (sometimes pseudostratified) primed epithelium (figure 1). In Rauber's layer species, the apical side of the epiblast thus always becomes exposed to the maternal environment. Later in development, after gastrulation has begun, this direct exposure to the maternal environment is terminated by the folding of the edges of the embryonic ectoderm (the ‘amniotic’ ectoderm) over the embryonic disc to form the enclosing amniotic cavity. This mode of amniotic cavity formation is shared by Rauber's layer species with the non-eutherian mammals and represents the ancestral mode of amnion formation also seen in non-mammalian Sauropsid amniotes, that is, birds and reptiles [59] (figure 2).

### (b) Species maintaining the polar trophoblast

In non-Rauber's layer species, the polar trophoblast is not only maintained until at least post-gastrulation stages but may be more proliferative than mural trophoblast.

Mice represent the most extreme example of such differential proliferation in that mural trophoblast cells undergo DNA endoreduplication to form polyploid giant cells, which are non-replicative, terminally differentiated, invasive trophoblast cells. Polar trophoblast cells, by contrast, maintain diploidy and proliferate rapidly into a mass of cells termed the extraembryonic ectoderm, that displaces the epiblast-spheroid into the blastocyst cavity to cause the embryo to assume an ‘egg-cylinder’ configuration [60]. The polar trophoblast is dependent for its proliferation on FGF4 and transforming growth factor beta (TGFβ)-like growth factors (probably nodal growth differentiation factor (NODAL)) secreted from the underlying naive epiblast [61–64]. Signalling downstream of FGF can be visualized via extracellular signal-regulated kinase (ERK) and AKT phosphorylation, and was found to be restricted to polar trophoblast [61]. An alternative assay, using mice genetically modified with an ERK-reporter, revealed a signalling gradient in the trophoblast, with highest activity in the polar region [65].

Similar to the mouse, in guinea pigs, belonging to the rodent suborder Hystricomorpha, the polar trophoblast requires signals from the epiblast to maintain cell division and to prevent premature differentiation into giant cells, as shown under *in vitro* culture conditions [66].

In higher primates (parvorder Catarrhini) such as humans and rhesus monkeys, both polar and mural trophoblast proliferates but is more intense on the epiblast-containing (embryonic) polar side [8,67,68]. In human embryos cultured in two- or three-dimensions, increasing distance from the OCT4-positive epiblast resulted in progressive differentiation of polar trophoblast-like cytotrophoblast cells into syncytiotrophoblast and then extravillous trophoblast cells [69–71]. This suggests that epiblast-derived signals prevent differentiation. The signals preventing such trophoblast terminal differentiation may include epidermal growth factor and WNT while TGFβ-like pathways may promote it, as suggested from *in vitro* studies aimed at isolating human trophoblast stem cells [72]. In both mice and humans, one target of such signals may be the transcription factor complex TEAD4/YAP1, which activates numerous cell cycle genes required for proliferation and once activated, can maintain its own transcription [73].

In the natal clinging bat (Miniopteridae), the California leaf-nosed bat and the long-tongued bat (Phyllostomidae), preferential proliferation is again seen in the trophoblast hemisphere containing the epiblast [32,74,75]. In armadillos, polar trophoblast proliferates, while mural trophoblast degenerates [76]. However, in this species, the proliferation of polar trophoblast may not depend on signals from the ICM, as evidenced by trophoblast mitotic figures seen in an embryo with a necrotic ICM [77].

In all of the above examples, the initial site of attachment of the blastocyst to the uterine epithelium and/or subsequent uterine invasion is on the more-proliferative polar trophoblast side of the embryo [59,78]. By contrast, most Rauber's layer mammals which lose their polar trophoblast implant on their abembryonic side or circumferentially [59]. In spite of this correlation, polar trophoblast maintenance is neither necessary for implantation on the embryonic side nor does it preclude abembryonic implantation:

(i) some Rauber's layer animals do implant on the embryonic side. Examples include Rauber's layer rodents such as kangaroo rats (Heteromyidae family) and squirrels (suborder Sciuromorpha) where implantation does occur on the side, where the polar trophoblast was [16], rabbits (Lagomorpha) and primitive primates such as lorises; and
(ii) in some species maintaining the polar trophoblast, uterine attachment is *not* on the embryonic side, but circumferential, i.e. also occurring on the mural trophoblast side, and thus likely to be independent of the polar trophoblast and of the underlying epiblast. Interestingly in these species, no preferential proliferation of polar trophoblast occurs relative to mural trophoblast. Examples are the short-tailed fruit bat (Phyllostomidae family) and the little brown bat (Vespertilionidae) [29,30].

While not necessary, the frequent correlation of maintenance of polar trophoblast—which often involves signalling from the underlying epiblast—with implantation occurring on the embryonic side, suggests that such an arrangement is of evolutionary benefit [15,78,79]. The benefit is likely to lie in the adaptation of novel modes of implantation. This may be best seen in primates and rodents. In the more ancestral species of these mammalian orders, polar trophoblast is lost, whereas in the more modern ones it is retained (figure 2), with a parallel trend for implantation on the abembryonic to embryonic (polar) side. With the embryonic-side implantation mode typical of polar trophoblast-retaining species, novel, more invasive (interstitial) modes of implantation became more prominent, as discussed eloquently for primates by James P. Hill in his Croonian lecture [10]. The driving force for implantation on the embryonic side is not known, but it may be that this is a particularly effective arrangement to bring the trophoblast-derived chorion closer to the allantoic vasculature that links the chorioallantoic placenta to the embryo.

## 4. Effects of the polar trophoblast on the epiblast

**Figure 3.**
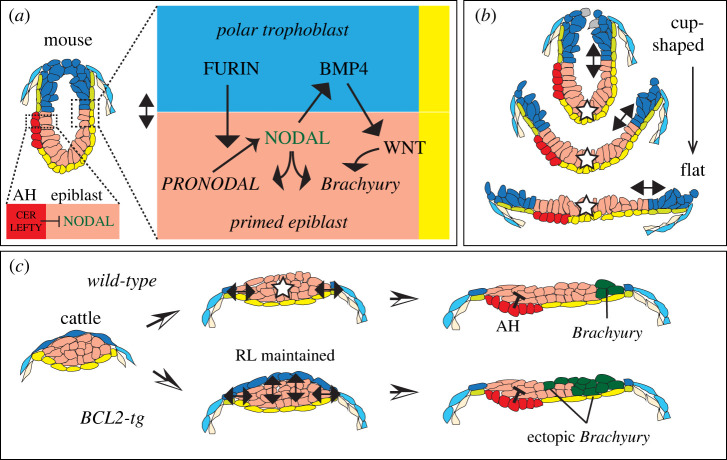
Trophoblast–epiblast signalling. (*a*) Signalling interactions in the mouse, between polar trophoblast and epithelial epiblast, leading to induction of *Brachyury* transcription at the rim of the cup-shaped epiblast (embryos drawn as saggital sections), close to the trophoblast border, where NODAL and WNT activities are the highest. NODAL is excluded from the future anterior via secretion from the anterior hypoblast (AH) of the CERBERUS and LEFTY inhibitors. (*b*) Imaginary ‘opening up’ of the cup-shaped mouse-like embryo leads to a Rauber's layer mammal-like flat epiblast (disc shaped if seen from above). Note that the distal epiblast is distanced and thus shielded (star) from the signal (double arrow) at the border and corresponds to the central epiblast of the flattened embryo. (*c*) Experimental set-up and result for maintaining Rauber's layer (RL) in cattle embryos by overexpressing BCL2 (BCL2-tg), leading to ectopic signalling and resultant overexpression of the gastrulation marker *Brachyury*.

Interactions that may occur between polar trophoblast and the underlying epiblast have been studied almost exclusively in mice. In mice not only is polar trophoblast proliferation dependent on signals from the epiblast (§3b), but the polar trophoblast reciprocally affects epiblast proliferation and patterning (figure 3*a*). The epiblast initially ubiquitously expresses mRNA coding for NODAL. NODAL is secreted as a less-active PRO-NODAL precursor and requires cleavage by the convertases FURIN or PACE4 to be fully active. These convertases diffuse into the epiblast from the trophoblast. As the diffusion distance of the convertases is limited, NODAL is activated most effectively closest to the epiblast–trophoblast border [80]. The proximal NODAL activation is then stabilized by an auto-regulatory loop involving a NODAL-responsive enhancer driving *NODAL* expression. NODAL causes increased epiblast proliferation but also diffuses into the adjacent polar trophoblast to maintain convertase expression and *BMP4* transcription [81]. Bone morphogenetic protein 4 (BMP4) from the polar trophoblast diffuses into the epiblast to turn on transcription of the NODAL cofactor *CRIPTO/TDGF1* [80] as well as *WNT* [81]. WNT, together with the activated NODAL, then switch on gastrulation genes such as *BRACHYURY/T* [82]. Importantly the reciprocal requirement for factors expressed only in adjacent tissues means that the end-outcome—in this case gastrulation induction—occurs at, and is dependent on, a boundary, in this case, the polar trophoblast–epiblast boundary. In the mouse, this boundary is circular, as both the epiblast and the polar trophoblast masses cavitate with the cavities subsequently joining [83], leaving only the rim of the cup-shaped epiblast touching the rim of the hollowed-out polar trophoblast. Gastrulation then occurs on only the posterior side of this rim due to the influence of NODAL, BMP and WNT inhibitors secreted from the anterior region of the enveloping hypoblast [84]. Importantly, the epiblast in the distal region of the egg cylinder (the base of the epiblast-cup) is protected from the inductive influences of the polar trophoblast, ensuring that gastrulation does not occur ectopically (figure 3*b*).

In human embryos, which also retain the polar trophoblast, some information has been gleaned from growing primed embryonic stem cells in two dimensions on micropatterned slides. While this system is a far cry from the *in vivo* situation, the same signalling pathways seen in the mouse—NODAL/WNT/BMP—were involved in turning on the mesoderm marker BRACHYURY [85]. In such systems, boundaries are created by differential display of signalling receptors at the colony edges while direct cell-cell contacts were essential for establishing diffusion-mediated cross-regulatory interactions to establish territories of differential marker expressions [86–88].

This gives rise to a pivotal question. Given the key involvement of the polar trophoblast in gastrulation induction in the mouse, how can this be reconciled with the loss of this very tissue in Rauber's layer mammals or indeed in the more ancestral mammalian groups such as monotremes and marsupials in which no polar trophoblast ever forms? The answer lies in the observation that in these species, the epithelialized epiblast disc establishes close contacts along its rim with the mural trophoblast (figure 3*b*). Thus a trophoblast–epiblast border with reciprocal interactions can be established, with the difference that the edge of the *mural* trophoblast takes over the function of the *polar* trophoblast. In line with this interpretation, in cattle, the polar as well as the mural trophoblast immediately adjacent to it, express FURIN, one of the important convertases restricting NODAL activity to the epiblast-trophoblast border [26,89]. This is also the case in other Rauber's layer species such as pigs, rabbits and the musk shrew (order Eulipotyphla) [35]. Indeed the progressive restriction of Nodal expression in the epiblast to the edge, as seen in the mouse, seems to be conserved in these four Rauber's layer mammals which are representative of three different mammalian orders [26,35,90,91]. Whether BMP signalling is also involved in gastrulation induction via the trophoblast–epiblast border is less clear, partly because there are several BMP paralogues (BMP2, 4, 8a, 8b) that could fulfil this function [26,35,52,92].

If we accept the premise that the trophoblast–epiblast border is involved in restricting gastrulation to the rim of the epiblast, it follows as a corollary that distancing of the polar trophoblast from the central ‘non-rim’ regions of the epiblast may be necessary to prevent widespread reciprocal interactions. This prediction was recently shown to be correct in a typical Rauber's layer species, namely in cattle [53]. The authors overexpressed BCL2, thereby reducing apoptosis, which delayed Rauber's layer disappearance by about a day. Intriguingly, this resulted in embryos ectopically expressing the primitive streak and mesoderm marker *BRACHYURY* in the epiblast, indicating the continuous presence, and thus signalling of the polar trophoblast overlying the central epiblast, was incompatible with normal development (figure 3*c*). While the causative Rauber's layer-epiblast signalling mechanism underlying the ectopic Brachyury induction was not determined, it is likely to involve some combination of the NODAL, WNT and BMP pathways as overstimulation in the mouse of NODAL [93], WNT [94,95] or BMP signalling [96] leads to ectopic *BRACHYURY* activation and/or supernumerary gastrulation initiation sites.

In summary, the polar trophoblast/Rauber's layer is able to communicate with the underlying epiblast, leading to gastrulation initiation. In mice, the everted cup-shape of the epiblast distances and thus shields the central/distal epiblast from the reciprocal trophoblast–epiblast interactions that lead to gastrulation. In Rauber's layer animals such as cattle, central epiblast shielding is automatically achieved as a consequence of removal of the polar trophoblast, again restricting trophoblast–epiblast interactions to the epiblast edge. Thus, paradoxically, the importance of Rauber's layer lies in its disappearance.

## 5. Loss of the polar trophoblast in relation to epiblast cavity formation

This brings us to a new question: if the central epiblast needs to be shielded from interactions with the trophoblast, how is this achieved in mammals (including humans) that maintain the polar trophoblast, but which, unlike the mouse, do not form a cup-shaped epiblast? A potential answer is that the epiblast cavitates (first proposed in [53]). By forming a cavity within the epiblast, the floor of the cavity, corresponding to the central epiblast, would be shielded from the polar trophoblast by the roof of the cavity as well as by the physical separation provided by the cavity itself. For this to be relevant requires that (i) cavitation is highly linked to polar trophoblast maintenance, (ii) cavitation is less well linked to alternative functions and (iii) cavitation can develop relatively easily, in line with the observation that a switch to polar trophoblast maintenance occurred repeatedly during evolution (§3).

### (a) Is epiblast cavitation correlated with polar trophoblast maintenance?

**Figure 4.**
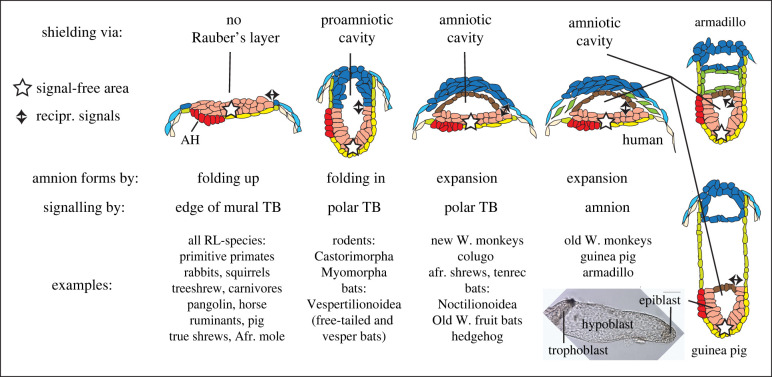
Methods of central epiblast shielding from extraembryonic (polar trophoblast or amniotic ectoderm) signals. It is proposed that central epiblast shielding can be achieved via removal of Rauber's layer (RL) or by the distancing of the central epiblast by formation of a proamniotic or amniotic cavity. In the latter case the possibility of the amniotic ectoderm (the roof of the epiblast cavity) substituting for the polar trophoblast or edge of the mural trophoblast to induce signalling leading to gastrulation seems particularly likely for the indicated species. The inset shows a 9-day old guinea pig embryo just prior to epiblast cavitation. Green-coloured cells in human and armadillo diagrams represent mesenchymal cells often termed mesodermal but likely to be of hypoblast origin. Afr., African; TB, trophoblast; W, World.

Interestingly, cavitation of the epiblast is not seen in the monotreme and marsupial sister-groups of eutherian mammals nor is it a feature of the pluripotent layers in evolutionary more distant amniotes [4,97–99]. Furthermore, nearly all Rauber's layer animals similarly do not form a cavity within the epiblast (figure 2) and those that do, such as cattle and pigs, form only a transient cavity or multiple small spaces between epiblast cells, which appears to be required for the transition to an epithelialized epiblast [26,27]. In notable contrast, ALL species that maintain the polar trophoblast, form a prominent cavity within the epiblast (figures 2 and 4).

### (b) Is amniotic formation the primary driver for epiblast cavitation?

In many trophoblast-maintaining species, the purpose of epiblast cavitation has always been ascribed to amnion formation, because the roof of the epiblast cavity expands to eventually form the inner ectodermal layer of the amniotic sac that envelops and protects the fetus from mechanical shock [15,59]. This type of epiblast cavity is thus termed the amniotic cavity (figure 1). However, in other polar trophoblast-maintaining species (mainly bats and rodents; see figures 1 and 2) a proamniotic cavity is formed as the roof cells of the initial epiblast cavity disappear, creating a cavity bounded by the epiblast ventrally and trophoblast dorsally. In these cases the true amniotic cavity is formed only after gastrulation has commenced via a folding process involving the margin of the epiblast [59,100]. Indeed, the folding mode of amnion formation, which is also used by all Rauber's layer mammals, is the ancestral one, and is also seen in non-placental mammals, birds and reptiles [101]. We can thus infer that epiblast cavitation has been adapted in some polar-trophoblast maintaining mammals as an alternate means of amnion formation. Note that this is a derived feature, a repurposing of a process that had evolved for a different reason.

### (c) Forming a cavity in the epiblast: how adaptable is the process?

Epiblast cavitation seems to have evolved from an inherent tendency of many types of epithelial cells to require a basement membrane for their survival [102–106]. Thus cells distant from a laminin-expressing membrane have to either reposition themselves or die, which can cause spaces and lumens to appear depending on the shape of the epithelializing epiblast (spheroid to flat ellipsoid). In humans and mice, central cavities are formed via a process termed hollowing that involves epitheloid epiblast cells organizing into radial rosettes followed by lumen formation [106,107]. Interestingly even embryonic stem cells in culture, embedded in a lamina-like matrix, exhibit this behaviour as they transit from a naive to a primed state [102,106,108]. In some bats (Noctilionidae, Phyllostomidae) the process is different. Small irregular spaces developing between epiblast cells coalesce, and extensive apoptosis is evident [28,29]. Interestingly, hollowing as well as apoptosis were seen in mouse embryos that had been manipulated to have twice the normal size [109]. This indicates that embryos can use both modes of cavitation (hollowing versus apoptosis) somewhat interchangeably depending on their morphology, indicative of the adaptability of the process of cavitation.

Cavitation is further adaptable as it is influenced not only by epiblast shape but also by the extent that the epiblast is covered by a laminin-containing basement membrane. In mammals, a basement membrane is formed between hypoblast and epiblast, though usually not between epiblast and trophoblast [51,67,102]. Hence, species where the hypoblast surrounds most of a sphere-shaped mass of epiblast cells would be predisposed to form a central lumen/amniotic cavity. Examples are polar trophoblast maintaining species such as armadillos [40], elephant and jumping shrews [39,49], tenrecs [37], colugos [12] and some bats of the Pteropodidae, Phyllostomidae and Thyropteridae families [28,110], where the hypoblast and lamina nearly completely envelop the epiblast (figure 2). Similarly in cultured human embryos laminin was seen to envelop the entire epiblast on day 8, just before amniotic cavity formation [71]. An evolutionarily distinct central lumen-generating variation is seen in rhesus macaque monkeys [9], the California leaf-nosed bat (Phyllostomidae) [74] and Noctilionidae bats [28], where a lamina additionally forms between some of the polar trophoblast and epiblast, thus extending the basal lamina beyond the hypoblast to encircle most of the epiblast, again resulting in amniotic cavity formation.

On the other hand, in species where the epiblast is only partially covered, a central lumen might be expected to break through the non-covered side to form a proamniotic cavity. This is seen in several rodents (e.g. mice, field voles; figure 1 on right), where the hypoblast is stretched only around the sides of the cup-shaped epiblast and the roof of the epiblast cup abuts the polar trophoblast with no basement membrane in between [14,111].

Conversely, no cavity is usually seen, nor would it be expected, in most Rauber's layer mammals, which generally have a flatter epiblast and a membrane-less close apposition to the polar trophoblast (§3a). In this situation epiblast cells can simply rearrange into an epithelium or pseudostratified epithelium guided by the underlying hypoblast-epiblast basement membrane with transient irregular cavities or spaces rarely—and then only very transiently—merging into a central cavity. One interesting transitional case is the tree shrew *Tupaia belangerie* which is a Rauber's layer species that has a near-spherical epiblast surrounded by hypoblast except for the dorsal side. During epithelialization this species develops a slit-like cavity in the centre at the same time that Rauber's layer disintegrates [19] (figure 2). The epiblast subsequently flattens out.

It can be concluded that cavitation could repeatedly arise in multiple mammalian species owing to: (i) the interchangeability in the mechanisms of cavity formation (apoptosis versus hollowing), and (ii) the multiple possibilities for adapting the epithelialization requirement for a lamina to generate a cavity, by modulating the shape of the epiblast and/or its membrane coverage.

### (d) A novel role for epiblast cavitation

Based on the above discussion, I propose (figure 4) that epiblast cavity formation is a mammalian prerequisite not primarily for amnion formation or epiblast epithelialization, but to allow embryos to maintain their polar trophoblast, thereby allowing them to explore new modes of implantation. In other words, the requirement for central epiblast shielding is evolutionarily closely coupled to epiblast cavity formation. Given that some polar trophoblast-maintaining species discard the roof of the epiblast cavity—forming a proamniotic cavity instead of an amniotic cavity—a corollary is that the cavity *per se*, as opposed to the roof of the cavity, is sufficient for providing shielding from the overlying trophoblast.

## 6. Variations on a theme—the amniotic ectoderm equivalence to the polar trophoblast

The idea that Rauber's layer disappearance and distancing of the polar trophoblast through epiblast cavitation simply represent two alternative mechanisms for restricting trophoblast signalling to the edge of the epiblast builds on the concept that boundaries between different tissues are critical for patterning. The reciprocal interaction between the extraembryonic trophoblast and the embryonic epiblast with factors required by one tissue originating from the neighbouring tissue ensures that patterning effects are realised only near the boundary, within the diffusion range of the signalling molecules [112,113].

However, there are mammals in which the epiblast-trophoblast boundary may not be involved at all in setting up a gastrulation-inducing circuit. The best example of this may be the guinea pig, in which the sphere of epiblast cells becomes physically distanced from the polar trophoblast by proliferation of the hypoblast (figure 4). This separation occurs well before gastrulation commences (figure 4). In spite of the absence of adjoining trophoblast, epiblast cavitation occurs, thus contradicting the idea that cavitation functions to shield the central epiblast from persisting polar trophoblast and indeed the very notion that trophoblast is involved in gastrulation induction at all!

A second example of trophoblast epiblast distancing occurring well before gastrulation commences is seen in the nine-banded armadillo, where a precocious exocoelum develops [76]. Mesenchymal cells of unknown origin line the inside of a cavity above the amniotic cavity—the outer walls of this cavity appear to be hypoblast as opposed to trophoblast (figure 4, rightmost).

A less drastic situation can be seen in humans and cynomolgus macaque monkeys where the hypoblast-derived extraembryonic mesenchymal cells interpose themselves between the (amniotic ectodermal) roof of the epiblast cavity and the polar (cyto-) trophoblast. In these instances, direct contact between trophoblast and epiblast may be broken before gastrulation can be induced (figure 4).

The mechanistic details of gastrulation induction in these species need to be examined in more detail. Increasing evidence suggests that, at least in humans and old world monkeys (Cercopithecidae), the roof of the amniotic cavity, comprising the amniotic ectoderm, can substitute for the polar trophoblast. The evidence supporting this idea is as follows:

(i) the gene expression profile of the epiblast roof (amniotic ectoderm) is distinct from the primed/epithelialized epiblast as shown in human and cynomolgus monkey pre-gastrulation embryos [71,114]. Thus the contact zone represents a bona fide boundary between alternative tissues;
(ii) the primate amniotic ectoderm resembles the mouse polar trophoblast. The mouse trophoblast stem cell markers *CDX2* and *GATA3* are expressed respectively in cynomolgus and human amniotic ectoderm [71,115]. Indeed, human trophoblast and amniotic cell identity have often been confused owing to a similar transcriptional profile [116]. Importantly, the NODAL activator FURIN is transcribed in human amniotic ectoderm, as indicated in the electronic supplementary material, table S2.1 of Xiang *et al.* [71]. The second trophoblast signal important for gastrulation induction in the mouse, namely BMP4, is expressed in the amniotic ectoderm of monkeys and humans [71,114]. Thus the amniotic ectoderm is in principle equipped to execute the reciprocal signalling shown to be important in the mouse polar trophoblast; and
(iii) the best evidence comes from an *in vitro* study, where human primed embryonic stem cells (ES) were first differentiated into amniotic ectoderm-like cells using BMP4, then BMP4 was withdrawn and the cells mixed with undifferentiated ES cells and cultured for 2 days [117]. This led to the induction of gastrulation markers, including BRACHYURY. These authors were also able to coax ES cells into epiblast cysts which in the presence of exogenous BMP4 formed sacs consisting of dorsal amniotic ectoderm and ventral epiblast epithelium. Interestingly these structures always commenced gastrulation at the boundary between these tissues, indicating that the ‘amniotic’ cavity is sufficient to shield the central epiblast from gastrulation-inducing effects.

Thus cavitation would still be required as a shielding mechanism but from the amniotic ectoderm instead of the polar trophoblast.

## 7. Conclusion

As mammals faced the selection pressures of alternative modes of implantation, they needed to adapt to novel requirements. The transition to close contact with the endometrium consequent to the loss of the egg shell may have necessitated the enclosing of the pluriblast within the trophoblast. However, the resulting apposition of the trophoblast with central epiblast presented a problem in terms of regulating the initiation of gastrulation. Two solutions to this are seen, to either lose the polar trophoblast again or to develop a cavity within the epiblast. Cavitation is a simple adaptation of inherent epithelialization processes coupled to epiblast shape and lamina coverage. Complete cavitation with the development of a persistent cavity roof allowed the secondment of the cavity roof in directly forming the amnion (as opposed to the ancestral folding process) as well as for gastrulation induction (and germ cell formation).

## Data Availability

This article has no additional data.

## References

[1] Rauber A. 1875 Die erste Entwicklung des Kaninchens. Sitzungsber. Naturfor. Gesell. Leipzig **10**, 103-109.

[2] Koellicker A. 1880 Die Entwickelung der Keimblatter des Kaninchens. Zool. Anz. **3**, 370-375.

[3] Frankenberg S, Shaw G, Freyer C, Pask AJ, Renfree MB. 2013 Early cell lineage specification in a marsupial: a case for diverse mechanisms among mammals. Development **140**, 965-975. (10.1242/dev.091629) 23344710

[4] Frankenberg S. 2018 Chapter Ten - Pre-gastrula development of non-eutherian mammals. In Current topics in developmental biology (eds B Plusa, A-K Hadjantonakis), pp. 237-266. New York, NY: Academic Press. 10.1016/bs.ctdb.2017.10.013 29477165

[5] Selwood L, Johnson MH. 2006 Trophoblast and hypoblast in the monotreme, marsupial and eutherian mammal: evolution and origins. BioEssays **28**, 128-145. (10.1002/bies.20360) 16435291

[6] Murphy WJ, Foley NM, Bredemeyer KR, Gatesy J, Springer MS. 2021 Phylogenomics and the genetic architecture of the placental mammal radiation. Annu. Rev. Anim. Biosci. **9**, 29-53. (10.1146/annurev-animal-061220-023149) 33228377

[7] Liu L et al. 2017 Genomic evidence reveals a radiation of placental mammals uninterrupted by the KPg boundary. Proc. Natl Acad. Sci. USA **114**, E7282. 28808022 10.1073/pnas.1616744114 PMC5584403

[8] Hertig AT, Rock J, Adams EC. 1956 A description of 34 human ova within the first 17 days of development. Am. J. Anat. **98**, 435-493. (10.1002/aja.1000980306) 13362122

[9] Enders AC, Schlafke S, Hendrickx AG. 1986 Differentiation of the embryonic disc, amnion, and yolk sac in the rhesus monkey. Am. J. Anat. **177**, 161-185. (10.1002/aja.1001770205) 3788819

[10] Hill JP. 1932 Croonian lecture: the developmental history of the primates. Phil. Trans. R. Soc. Lond. B Contain. Pap. Biol. Character (1896–1934) **221**, 45-178. (10.1098/rstb.1932.0002)

[11] Hubrecht AAW. 1902 Furchung und keimblattbildung bei tarsius spectrum. Amsterdam, The Netherlands: Johannes Mueller.

[12] Hubrecht AAW, De Lange D. 1919 Früheste Entwicklungsstadien und Placentation von Galeopithecus. Verh. K. Akad. Wet. te Amst. **2**, 1-39.

[13] Williams BS, Biggers JD. 1990 Polar trophoblast (Rauber's layer) of the rabbit blastocyst. Anat. Rec. **227**, 211-222. (10.1002/ar.1092270210) 2350010

[14] Kaufman MH. 1995 The atlas of mouse development. London, UK: Academic Press.

[15] Mossman HW. 1937 Comparative morphogenesis of the fetal membranes and accessory uterine structures. Contrib. Embryol. **26**, 133-137. 10.1016/0143-4004(91)90504-9 2034592

[16] Nielson PE. 1940 The fetal membranes of the kangaroo rat, dipodomys, with a consideration of the phylogeny of the Geomyoidea. Anat. Rec. **77**, 103-127. (10.1002/ar.1090770110)

[17] Duval M. 1892 Le Placenta des Rongeurs. Le placenta du cochon d'Inde. J. Anat. Physiol. **28**, 58-98.

[18] Volker O. 1908 Ueber die ersten Entwicklungsvorgaenge beim Ziesel. Anat. Anz. **33**, 98-111.

[19] Kuhn H-J, Schwaier A. 1973 Implantation, early placentation, and the chronology of embryogenesis in Tupaia belangeri. Z. Anat. Entwicklungsgeschichte **142**, 315-340. (10.1007/BF00519135) 4798750

[20] Hill JP, Tribe M. 1924 The early development of the cat (*Felis domestica*). Q. J. Microsc. Sci. **68**, 513-U514.

[21] Van der Stricht O. 1923 The blastocyst of the dog. J. Anat. **58**, 52-53. 17103995 PMC1249776

[22] Enders AC. 1986 Morphological changes in the blastocyst of the western spotted skunk during activation from delayed implantation. Biol. Reprod. **34**, 423-437. (10.1095/biolreprod34.2.423) 3955151

[23] Hamilton WJ. 1934 XI.—The early stages in the development of the ferret: fertilisation to the formation of the prochordal plate. Trans. R. Soc. Edinb. **58**, 251-278. (10.1017/S0080456800023127)

[24] de Lange D. 1933 Placentarbildung. In Handbuch der vergleichended anatomie der wirbeltiere (eds L Bolk, E Göppert, E Kallius, W Lubosch), pp. 155-234. Berlin, Germany: Urban und Schwarzenberg.

[25] Enders AC, Lantz KC, Liu IK, Schlafke S. 1988 Loss of polar trophoblast during differentiation of the blastocyst of the horse. J. Reprod. Fertil. **83**, 447-460. (10.1530/jrf.0.0830447) 3397953

[26] van Leeuwen J, Berg DK, Pfeffer PL. 2015 Morphological and gene expression changes in cattle embryos from hatched blastocyst to early gastrulation stages after transfer of *in vitro* produced embryos. PLoS ONE **10**, e0129787. (10.1371/journal.pone.0129787) 26076128 PMC4468082

[27] Harmoush B, Tsikolia N, Viebahn C. 2021 Epiblast and trophoblast morphogenesis in the pre-gastrulation blastocyst of the pig. A light- and electron-microscopical study. J. Morphol. **282**, 1339-1361. (10.1002/jmor.21389) 34176156

[28] Rasweiler JJt, Badwaik NK. 1996 Unusual aspects of inner cell mass formation, endoderm differentiation, Reichert's membrane development, and amniogenesis in the lesser bulldog bat, *Noctilio albiventri*. Anat. Rec. **246**, 293-304. (10.1002/(SICI)1097-0185(199610)246:2<293::AID-AR15>3.0.CO;2-J) 8888970

[29] Badwaik NK, Rasweiler JJt, Oliveira SF. 1997 Formation of reticulated endoderm, Reichert's membrane, and amniogenesis in blastocysts of captive-bred, short-tailed fruit bats, *Carollia perspicillata*. Anat. Rec. **247**, 85-101. (10.1002/(SICI)1097-0185(199701)247:1<85::AID-AR11>3.0.CO;2-6) 8986306

[30] Wimsatt WA. 1944 An analysis of implantation in the bat *Myotis lucifugus lucifugus*. Am. J. Anat. **74**, 355-411. (10.1002/aja.1000740304)

[31] Van der Stricht O. 1899 La fixation de l'oeuf de chauve-souris a l'interieur de l'uterus (V. noctula). Verh. Anatoischen Ges. **13**, 76-95.

[32] van der Merwe M. 1982 Histological study of implantation in the natal clinging bat (*Miniopterus schreibersii natalensis*). J. Reprod. Fertil. **65**, 319-323. (10.1530/jrf.0.0650319) 7097640

[33] Heideman PD, Powell KS. 1998 Age-specific reproductive strategies and delayed embryonic development in an old world fruit bat, *Ptenochirus jagori*. J. Mammal. **79**, 295-311. (10.2307/1382866)

[34] Hubrecht AAW. 1889 Studies in mammalian embryology. I. The placentation of *Erinaceus europaeus*, with remarks on the phylogeny of the placenta. Q. J. Micr. Sci. **30**, 283-404.

[35] Yoshida M, Kajikawa E, Kurokawa D, Tokunaga T, Onishi A, Yonemura S, Kobayashi K, Kiyonari H, Aizawa S. 2016b Conserved and divergent expression patterns of markers of axial development in eutherian mammals. Dev. Dyn. **245**, 67-86. (10.1002/dvdy.24352) 26404161

[36] Gabie V. 1959 The early embryology of *Eremitalpa granti* (Broom). J. Morphol. **104**, 181-203. (10.1002/jmor.1051040202) 13825807

[37] Goetz RH. 1938 Early development of the Tenrecoidea. BioMorphosis **1**, 67-79.

[38] van der Horst CJ. 1949 The placentation of *Elephantulus*. Trans. R. Soc. S. Afr. **32**, 435-629. (10.1080/00359194909519872)

[39] De Lange D. 1949 Communication on the attachment and the early development of macroscelides (=*Elephantulus*) Rozeti duv., The North-African jumping shrew. Bijdragen tot de Dierkunde **28**, 255-285. (10.1163/26660644-02801033)

[40] Patterson JT. 1913 *Polyembryonic development in* Tatusia novemcincta. *J. Morphol*. **24**, 559-683.

[41] Frankenberg SR, De Barros FRO, Rossant J, Renfree MB. 2016 The mammalian blastocyst. WIREs Dev. Biol. **5**, 210-232. (10.1002/wdev.220) 26799266

[42] Chazaud C, Yamanaka Y, Pawson T, Rossant J. 2006 Early lineage segregation between epiblast and primitive endoderm in mouse blastocysts through the Grb2-MAPK pathway. Dev. Cell **10**, 615-624. (10.1016/j.devcel.2006.02.020) 16678776

[43] Piliszek A, Madeja ZE, Plusa B. 2017 Suppression of ERK signalling abolishes primitive endoderm formation but does not promote pluripotency in rabbit embryo. Development **144**, 3719-3730. 28935706 10.1242/dev.156406 PMC5675450

[44] Kuijk EW, van Tol LT, Van de Velde H, Wubbolts R, Welling M, Geijsen N, Roelen BA. 2012 The roles of FGF and MAP kinase signaling in the segregation of the epiblast and hypoblast cell lineages in bovine and human embryos. Development **139**, 871-882. (10.1242/dev.071688) 22278923 PMC3274353

[45] Rodríguez A, Allegrucci C, Alberio R. 2012 Modulation of pluripotency in the porcine embryo and iPS cells. PLoS ONE **7**, e49079. (10.1371/journal.pone.0049079) 23145076 PMC3493503

[46] Boroviak T, Loos R, Lombard P, Okahara J, Behr R, Sasaki E, Nichols J, Smith A, Bertone P. 2015 Lineage-specific profiling delineates the emergence and progression of naive pluripotency in mammalian embryogenesis. Dev. Cell **35**, 366-382. (10.1016/j.devcel.2015.10.011) 26555056 PMC4643313

[47] Niakan KK, Eggan K. 2013 Analysis of human embryos from zygote to blastocyst reveals distinct gene expression patterns relative to the mouse. Dev. Biol. **375**, 54-64. (10.1016/j.ydbio.2012.12.008) 23261930

[48] Pfeffer PL. 2018 Building principles for constructing a mammalian blastocyst embryo. Biology **7**, 41. (10.3390/biology7030041) 30041494 PMC6164496

[49] Van der Horst CJ. 1942 Early stages in the embryonic development of *Elephantulus*. S. Afr. J. Med. Sci. **7**, 55-67.

[50] Barends PM, Stroband HW, Taverne N, te Kronnie G, Leen MP, Blommers PC. 1989 Integrity of the preimplantation pig blastocyst during expansion and loss of polar trophectoderm (Rauber cells) and the morphology of the embryoblast as an indicator for developmental stage. J. Reprod. Fertil. **87**, 715-726. (10.1530/jrf.0.0870715) 2600919

[51] Vejlsted M, Avery B, Schmidt M, Greve T, Alexopoulos N, Maddox-Hyttel P. 2005 Ultrastructural and immunohistochemical characterization of the bovine epiblast. Biol. Reprod. **72**, 678-686. (10.1095/biolreprod.104.034348) 15537864

[52] Valdez Magana G, Rodriguez A, Zhang H, Webb R, Alberio R. 2014 Paracrine effects of embryo-derived FGF4 and BMP4 during pig trophoblast elongation. Dev. Biol. **387**, 15-27. (10.1016/j.ydbio.2014.01.008) 24445281

[53] Van Leeuwen J, Rawson P, Berg DK, Wells DN, Pfeffer PL. 2020 On the enigmatic disappearance of Rauber's layer. Proc. Natl Acad. Sci. USA **117**, 16 409-16 417. (10.1073/pnas.2002008117) PMC7368265 32601185

[54] Betteridge KJ, Flechon JE. 1988 The anatomy and physiology of pre-attachment bovine embryos. Theriogenology **29**, 155-187. (10.1016/0093-691X(88)90038-6)

[55] Talbot NC, Garrett WM. 2001 Ultrastructure of the embryonic stem cells of the 8-day pig blastocyst before and after *in vitro* manipulation: development of junctional apparatus and the lethal effects of PBS mediated cell-cell dissociation. Anat. Rec. **264**, 101-113. (10.1002/ar.1141) 11505376

[56] Kalkan T, Smith A. 2014 Mapping the route from naive pluripotency to lineage specification. Phil. Trans. R. Soc. B **369**, 20130540. (10.1098/rstb.2013.0540) PMC4216463 25349449

[57] Sheng G. 2015 Epiblast morphogenesis before gastrulation. Dev. Biol. **401**, 17-24. 10.1016/j.ydbio.2014.10.003 25446532

[58] Nichols J, Smith A. 2009 Naive and primed pluripotent states. Cell Stem Cell **4**, 487-492. (10.1016/j.stem.2009.05.015) 19497275

[59] Mossman HW. 1987 Vertebrate fetal membranes: comparative ontology and morphology; evolution; phylogenetic significance; basic functions; research opportunities. London/New Brunswick, NJ: Macmillan/Rutgers University Press.

[60] Weberling A, Zernicka-Goetz M. 2021 Trophectoderm mechanics direct epiblast shape upon embryo implantation. Cell Rep. **34**, 108655. (10.1016/j.celrep.2020.108655) 33472064 PMC7816124

[61] Christodoulou N, Weberling A, Strathdee D, Anderson KI, Timpson P, Zernicka-Goetz M. 2019 Morphogenesis of extra-embryonic tissues directs the remodelling of the mouse embryo at implantation. Nat. Commun. **10**, 3557. (10.1038/s41467-019-11482-5) PMC6686005 31391456

[62] Erlebacher A, Price KA, Glimcher LH. 2004 Maintenance of mouse trophoblast stem cell proliferation by TGF-beta/activin. Dev. Biol. **275**, 158-169. (10.1016/j.ydbio.2004.07.032) 15464579

[63] Gardner RL, Johnson MH. 1972 An investigation of inner cell mass and trophoblast tissues following their isolation from the mouse blastocyst. J. Embryol. Exp. Morphol. **28**, 279-312. 4672104

[64] Tanaka S, Kunath T, Hadjantonakis AK, Nagy A, Rossant J. 1998 Promotion of trophoblast stem cell proliferation by FGF4. Science **282**, 2072-2075. (10.1126/science.282.5396.2072) 9851926

[65] Simon CS, Rahman S, Raina D, Schröter C, Hadjantonakis A-K. 2020 Live visualization of ERK activity in the mouse blastocyst reveals lineage-specific signaling dynamics. Dev. Cell **55**, 341-353.e345. (10.1016/j.devcel.2020.09.030) 33091370 PMC7658048

[66] Ilgren EB. 1980 The control of trophoblastic growth in the guinea pig. J. Embryol. Exp. Morphol. **60**, 405-418. 7310279

[67] Enders AC, Schlafke S. 1981 Differentiation of the blastocyst of the rhesus monkey. Am. J. Anat. **162**, 1-21. (10.1002/aja.1001620102) 7304472

[68] Luckett WP. 1978 Origin and differentiation of the yolk sac and extraembryonic mesoderm in presomite human and rhesus monkey embryos. Am. J. Anat. **152**, 59-97. (10.1002/aja.1001520106) 98035

[69] Deglincerti A, Croft GF, Pietila LN, Zernicka-Goetz M, Siggia ED, Brivanlou AH. 2016 Self-organization of the *in vitro* attached human embryo. Nature **533**, 251-254. (10.1038/nature17948) 27144363

[70] West RC et al. 2019 Dynamics of trophoblast differentiation in peri-implantation–stage human embryos. Proc. Natl Acad. Sci. USA **116**, 22 635-22 644. (10.1073/pnas.1911362116) PMC6842583 31636193

[71] Xiang L et al. 2020 A developmental landscape of 3D-cultured human pre-gastrulation embryos. Nature **577**, 537-542. (10.1038/s41586-019-1875-y) 31830756

[72] Okae H et al. 2018 Derivation of human trophoblast stem cells. Cell Stem Cell **22**, 50-63.e56. (10.1016/j.stem.2017.11.004) 29249463

[73] Saha B et al. 2020 TEAD4 ensures postimplantation development by promoting trophoblast self-renewal: an implication in early human pregnancy loss. Proc. Natl Acad. Sci. USA **117**, 17 864-17 875. (10.1073/pnas.2002449117) PMC7395512 32669432

[74] Bleier WJ. 1975 Early embryology and implantation in the california leaf-nosed bat. Macrotus californicus. Anat. Rec. **182**, 237-253. 10.1002/ar.1091820208 1167300

[75] Rasweiler JJ. 1974 Reproduction in the long-tongued bat, *Glossophaga soricina*. II. Implantation and early embryonic development. Am. J. Anat. **139**, 1-35. (10.1002/aja.1001390102) 4810014

[76] Enders AC. 2002 Implantation in the nine-banded armadillo: how does a single blastocyst form four embryos? Placenta **23**, 71-85. (10.1053/plac.2001.0753) 11869094

[77] Enders AC. 1962 The structure of the armadillo blastocyst. J. Anat. **96**, 39-48. 13890166 PMC1244171

[78] Wimsatt WA. 1975 Some comparative aspects of implantation. Biol. Reprod. **12**, 1-40. (10.1095/biolreprod12.1.1) 806310

[79] Mossman HW. 1971 Orientation and site of attachment of the blastocyst: a comparative study. In The biology of the blastocyst (eds R.J Blandau), pp. 49-57. Chicago, IL: University of Chicago Press.

[80] Beck S, Le Good JA, Guzman M, Ben Haim N, Roy K, Beermann F, Constam DB. 2002 Extraembryonic proteases regulate nodal signalling during gastrulation. Nat. Cell Biol. **4**, 981-985. (10.1038/ncb890) 12447384

[81] Ben-Haim N, Lu C, Guzman-Ayala M, Pescatore L, Mesnard D, Bischofberger M, Naef F, Robertson EJ, Constam DB. 2006 The nodal precursor acting via activin receptors induces mesoderm by maintaining a source of its convertases and BMP4. Dev. Cell **11**, 313-323. (10.1016/j.devcel.2006.07.005) 16950123

[82] Yamaguchi TP, Takada S, Yoshikawa Y, Wu N, McMahon AP. 1999 T (Brachyury) is a direct target of Wnt3a during paraxial mesoderm specification. Genes Dev. **13**, 3185-3190. (10.1101/gad.13.24.3185) 10617567 PMC317203

[83] Christodoulou N, Kyprianou C, Weberling A, Wang R, Cui G, Peng G, Jing N, Zernicka-Goetz M. 2018 Sequential formation and resolution of multiple rosettes drive embryo remodelling after implantation. Nat. Cell Biol. **20**, 1278-1289. (10.1038/s41556-018-0211-3) 30323188

[84] Takaoka K, Hamada H. 2012 Cell fate decisions and axis determination in the early mouse embryo. Development **139**, 3-14. (10.1242/dev.060095) 22147950

[85] Warmflash A, Sorre B, Etoc F, Siggia ED, Brivanlou AH. 2014 A method to recapitulate early embryonic spatial patterning in human embryonic stem cells. Nat. Methods **11**, 847-854. (10.1038/nmeth.3016) 24973948 PMC4341966

[86] Chhabra S, Liu L, Goh R, Kong X, Warmflash A. 2019 Dissecting the dynamics of signaling events in the BMP, WNT, and NODAL cascade during self-organized fate patterning in human gastruloids. PLoS Biol. **17**, e3000498. (10.1371/journal.pbio.3000498) 31613879 PMC6814242

[87] Etoc F, Metzger J, Ruzo A, Kirst C, Yoney A, Ozair MZ, Brivanlou AH, Siggia ED. 2016 A balance between secreted inhibitors and edge sensing controls gastruloid self-organization. Dev. Cell. **39**, 302–315. 10.1016/j.devcel.2016.09.016 PMC5113147 27746044

[88] Martyn I, Kanno TY, Ruzo A, Siggia ED, Brivanlou AH. 2018 Self-organization of a human organizer by combined Wnt and nodal signalling. Nature **558**, 132-135. (10.1038/s41586-018-0150-y) 29795348 PMC6077985

[89] Degrelle SA, Campion E, Cabau C, Piumi F, Reinaud P, Richard C, Renard JP, Hue I. 2005 Molecular evidence for a critical period in mural trophoblast development in bovine blastocysts. Dev. Biol. **288**, 448-460. (10.1016/j.ydbio.2005.09.043) 16289134

[90] Hassoun R, Schwartz P, Feistel K, Blum M, Viebahn C. 2009 Axial differentiation and early gastrulation stages of the pig embryo. Differentiation **78**, 301-311. (10.1016/j.diff.2009.07.006) 19683851

[91] Ploger R, Viebahn C. 2018 Pitx2 and nodal as conserved early markers of the anterior-posterior axis in the rabbit embryo. Ann. Anat.=Anatomischer Anzeiger : official organ of the Anatomische Gesellschaft **218**, 256-264. (10.1016/j.aanat.2018.02.016) 29705588

[92] Hopf C, Viebahn C, Puschel B. 2011 BMP signals and the transcriptional repressor BLIMP1 during germline segregation in the mammalian embryo. Dev. Genes Evol. **221**, 209-223. (10.1007/s00427-011-0373-5) 21881976 PMC3192270

[93] Perea-Gomez A et al. 2002 Nodal antagonists in the anterior visceral endoderm prevent the formation of multiple primitive streaks. Dev. Cell **3**, 745-756. (10.1016/S1534-5807(02)00321-0) 12431380

[94] Merrill BJ, Pasolli HA, Polak L, Rendl M, García-García MJ, Anderson KV, Fuchs E. 2004 Tcf3: a transcriptional regulator of axis induction in the early embryo. Development **131**, 263-274. (10.1242/dev.00935) 14668413

[95] Popperl H, Schmidt C, Wilson V, Hume CR, Dodd J, Krumlauf R, Beddington RS. 1997 Misexpression of Cwnt8C in the mouse induces an ectopic embryonic axis and causes a truncation of the anterior neuroectoderm. Development **124**, 2997-3005. (10.1242/dev.124.15.2997) 9247341

[96] Pereira PN et al. 2012 Antagonism of Nodal signaling by BMP/Smad5 prevents ectopic primitive streak formation in the mouse amnion. Development **139**, 3343-3354. (10.1242/dev.075465) 22912414

[97] Voiculescu O, Bertocchini F, Wolpert L, Keller RE, Stern CD. 2007 The amniote primitive streak is defined by epithelial cell intercalation before gastrulation. Nature **449**, 1049-1052. (10.1038/nature06211) 17928866

[98] Yoshida M, Kajikawa E, Kurokawa D, Noro M, Iwai T, Yonemura S, Kobayashi K, Kiyonari H, Aizawa S. 2016a Conserved and divergent expression patterns of markers of axial development in reptilian embryos: Chinese soft-shell turtle and Madagascar ground gecko. Dev. Biol. **415**, 122-142. (10.1016/j.ydbio.2016.05.005) 27174471

[99] Yoshida M, Kajikawa E, Yamamoto D, Kurokawa D, Yonemura S, Kobayashi K, Kiyonari H, Aizawa S. 2016c Conserved and divergent expression patterns of markers of axial development in the laboratory opossum, *Monodelphis domestica*. Dev. Dyn. **245**, 1176-1188. (10.1002/dvdy.24459) 27666927

[100] Badwaik NK, Rasweiler JJ. 2000 6 - Pregnancy. In Reproductive biology of bats (eds EG Crichton, PH Krutzsch), pp. 221-293. London, UK: Academic Press.

[101] Mess A, Carter AM. 2006 Evolutionary transformations of fetal membrane characters in Eutheria with special reference to Afrotheria. J. Exp. Zool. B: Mol. Dev. Evol. **306B**, 140-163. 10.1002/jez.b.21079 16254985

[102] Bedzhov I, Zernicka-Goetz M. 2014 Self-organizing properties of mouse pluripotent cells initiate morphogenesis upon implantation. Cell **156**, 1032-1044. (10.1016/j.cell.2014.01.023) 24529478 PMC3991392

[103] Frisch S, Francis H. 1994 Disruption of epithelial cell-matrix interactions induces apoptosis. J. Cell Biol. **124**, 619-626. (10.1083/jcb.124.4.619) 8106557 PMC2119917

[104] Li S, Edgar D, Fässler R, Wadsworth W, Yurchenco PD. 2003 The role of laminin in embryonic cell polarization and tissue organization. Dev. Cell **4**, 613-624. (10.1016/S1534-5807(03)00128-X) 12737798

[105] Murray P, Edgar D. 2001 The regulation of embryonic stem cell differentiation by leukaemia inhibitory factor (LIF). Differentiation **68**, 227-234. (10.1046/j.1432-0436.2001.680410.x) 11776475

[106] Shahbazi MN et al. 2017 Pluripotent state transitions coordinate morphogenesis in mouse and human embryos. Nature **552**, 239-243. (10.1038/nature24675) 29186120 PMC5768241

[107] Kim YS, Fan R, Kremer L, Kuempel-Rink N, Mildner K, Zeuschner D, Hekking L, Stehling M, Bedzhov I. 2021 Deciphering epiblast lumenogenesis reveals proamniotic cavity control of embryo growth and patterning. Sci. Adv. **7**, eabe1640. (10.1126/sciadv.abe1640) 33692105 PMC7946377

[108] Taniguchi K et al. 2015 Lumen formation is an intrinsic property of isolated human pluripotent stem cells. Stem Cell Rep. **5**, 954-962. (10.1016/j.stemcr.2015.10.015) PMC4682207 26626176

[109] Orietti LC, Rosa VS, Antonica F, Kyprianou C, Mansfield W, Marques-Souza H, Shahbazi MN, Zernicka-Goetz M. 2021 Embryo size regulates the timing and mechanism of pluripotent tissue morphogenesis. Stem Cell Rep. **16**, 1182-1196. (10.1016/j.stemcr.2020.09.004) PMC8185375 33035465

[110] Wimsatt WA, Enders AC. 1980 Structure and morphogenesis of the uterus, placenta, and paraplacental organs of the neotropical disc-winged bat *Thyroptera tricolor spix* (Microchiroptera: Thyropteridae). Am. J. Anat. **159**, 209-243. (10.1002/aja.1001590208) 7446448

[111] Copp AJ, Clarke JR. 1988 Role of the polar trophectoderm in determining the pattern of early post-implantation morphogenesis in mammals: evidence from development of the short-tailed field vole, *Microtus agrestis*. Placenta **9**, 643-653. (10.1016/0143-4004(88)90008-2) 3070537

[112] Davidson EH. 2010 Emerging properties of animal gene regulatory networks. Nature **468**, 911-920. (10.1038/nature09645) 21164479 PMC3967874

[113] Gurdon JB, Harger P, Mitchell A, Lemaire P. 1994 Activin signalling and response to a morphogen gradient. Nature **371**, 487-492. 7935761 10.1038/371487a0

[114] Sasaki K et al. 2016 The germ cell fate of cynomolgus monkeys is specified in the nascent amnion. Dev. Cell **39**, 169-185. (10.1016/j.devcel.2016.09.007) 27720607

[115] Niu Y et al. 2019 Dissecting primate early post-implantation development using long-term *in vitro* embryo culture. Science **366**, eaaw5754. (10.1126/science.aaw5754) 31672917

[116] Guo G et al. 2021 Human naive epiblast cells possess unrestricted lineage potential. Cell Stem Cell **28**, 1040-1056.e1046. (10.1016/j.stem.2021.02.025) 33831366 PMC8189439

[117] Zheng Y et al. 2019 Controlled modelling of human epiblast and amnion development using stem cells. Nature **573**, 421-425. (10.1038/s41586-019-1535-2) 31511693 PMC8106232

